# Analysis of the Differential Efficacy of the Reduced Version Over the Extended Version of an Affective-Sexual Education Program for Adults with Intellectual Disabilities

**DOI:** 10.1007/s10508-022-02407-3

**Published:** 2022-09-28

**Authors:** María Dolores Gil-Llario, Olga Fernández-García, Tania B. Huedo-Medina, Verónica Estruch-García, Rafael Ballester-Arnal

**Affiliations:** 1 Department of Developmental and Educational Psychology, Faculty of Psychology, University of Valencia, Av. Blasco Ibáñez, 21, 46010 Valencia, Spain; 2 Department of Allied Health Sciences, College of Agriculture, Health, and Natural Resources, University of Connecticut, Storrs, CT USA; 3 Department of Basic and Clinical Psychology and Psychobiology, Faculty of Health Sciences, Jaume I University, Castellón de la Plana, Spain

**Keywords:** Affective-Sexual Education Program, Intellectual disabilities, Adults, Effectiveness

## Abstract

Recently, with the increase in demand, multiple intervention proposals aimed at improving the sexual health of people with intellectual disabilities have emerged. Among them is the SALUDIVERSEX program, which takes a positive approach to sexuality. It has an extended version, consisting of 16 sessions and whose efficacy has already been proven, and a reduced version of 10 sessions. Thus, the present study aimed to test the differential efficacy of the two versions. A total of 208 participants (103 women and 105 men) aged between 19 and 67 years (*M* = 37.23, SD = 10.66) completed a battery of instruments before and after the intervention. Statistical analyses showed that users who participated in the reduced version of the program presented a significantly higher rate of improvement in their sexual behaviors compared to those who participated in the extended version (Sexual response: *β*_10_ = − 0.46 ± 0.19, *p* = .034; Sex practices: *β*_10_ = − 0.52 ± 0.23, *p* = .037; Use condoms: *β*_10_ = − 1.56 ± 0.59, *p* = .017), as well as a significantly higher decrease in the risk of suffering sexual abuse (*β*_10_ = 3.95 ± 0.64, *p* < .001). However, no statistically significant differences in sexuality knowledge were obtained with respect to the improvement between the two versions (*β*_10_ = − 0.09 ± 1.21, *p* = .94). Meanwhile, the professionals who applied the program found that those who participated in the reduced version, although they presented a significantly greater increase in their knowledge about privacy (*β*_10_ = − 0.48 ± 0.08, *p* < .001), did not improve their concerns about their inappropriate sexual behaviors as much as the users of the extended version (*β*_10_ = − 1.35 ± 0.21, *p* < .001). Thus, although both versions were effective, the reduced version seems to do so to a greater extent and in a shorter time, which makes it the more recommendable option.

## Introduction

The affective-sexual education of people with intellectual disabilities (ID) has gone from being a conflictive aspect, due to the controversial attitudes of family members and professionals, to become an increasingly demanded aspect not only by the users themselves but also by the centers they attend (Yildiz & Cavkaytar, 2017). Thus, recent studies show low numbers in terms of the degree of knowledge and high percentages of demand for education on the part of users. For example, Kijak (2011) found that 90% of the young people with ID he assessed were not able to correctly explain what contraception was, what it is for, or name contraceptive methods; only 2% knew what sexually transmitted diseases were and could list at least two of them; and only 37% correctly defined the pubertal changes of the opposite sex. For their part, Lockhart et al. (2010) in their study with adults with ID from two large ID service providers in the greater Dublin area found that 80% did not know what menstruation was, 50% were not able to correctly identify different sexual practices, 75% did not know what orgasm was, and 46% showed incomplete knowledge about personal limits. More recently, Gil-Llario (2019), who analyzed knowledge about sexuality and vulnerability to sexual abuse in a sample of young adults with ID, detected that 51% believed that an acquaintance could not be a perpetrator of sexual abuse and 63% that at home it is impossible for someone to sexually abuse you. In this regard, it is assumed that the figures on the prevalence of sexual abuse are underrepresented for various reasons, including the fact that many victims do not report it because they are not aware that they are victims (Gil-Llario et al., 2021a). However, affective-sexual education not only has the function of promoting self-protection, but it must also be understood in a positive way. The proof is in the studies that find a relationship with the quality of life of the users (Gil-Llario, 2019; Morales et al., 2016; Rushbrooke et al., 2014).

With regard to residences and occupational centers for people with ID, it has been found that in those where an effort has been made to provide the necessary affective-sexual education, the number of conflicts between users has been reduced. In the centers, there are people with lower support needs who may abuse others who require more support. For example, a person with Prader–Willi may accept the demand of another user with a lesser degree of impairment at the adaptive and conceptual level, but with a repressed sexual orientation, to perform fellatio in exchange for food (Gil-Llario et al., 2021a).

For all these reasons, intervention proposals have recently emerged aimed at covering the key knowledge, attitudes and skills they need to know in order to develop a healthy sexual life (Aceldo et al., 2006; Gutiérrez-Bermejo et al., 2021; López, 2011; Navarro et al., 2010; Parra & Oliva, 2015; Rodriguez-Mayoral et al., 2006; van den Toren et al., 2021). Ideally, such proposals should understand sexuality as an aspect that transcends genitality and involves interaction with other people, therefore, including training on sexual communication and clearly establishing the boundaries of what is public and what is private in their specific cultural context will be essential, with special emphasis on indicators of sexual abuse and offering clear models of autoerotic sexual practices or sexual exchange (Gil-Llario et al., 2019). For example, people with ID often have difficulty initiating or ending interpersonal relationships and/or accepting or rejecting sexual relationships assertively (Schaafsma et al., 2017). This implies that educational strategies should go beyond the prevention of unwanted sexual behaviors or risky sexual practices, adopting a positivist perspective on sexuality. The aim is to transmit all learning by focusing on the acceptance of sexuality and sexual expression of all individuals, regardless of their characteristics, and conceiving it as something natural and healthy (Emerson, 2022). This will help people with ID to understand that sexuality is something inherent to every human being and that it goes beyond sexual practices. In addition, this avoids fear-based learning and denial, so common in this group, and encourages the assimilation of positive thoughts about sexuality (e.g., do not prohibit the performance of autoerotic practices, but inform them where and when they can be performed).

As far as the Spanish context is concerned, most of these proposals are general guidelines or recommendations that, despite offering very interesting resources, do not provide evidence of their validity (Aceldo et al., 2006; Barger et al., 2009; Gonzálvez et al., 2018). There are also some studies that do contemplate this need, although they focus on the objective of improving knowledge about a small number of topics such as those related to AIDS and the prevention of unwanted pregnancies, mainly talking about contraceptive methods. An example of this is the program developed by Aceldo et al. (2006). Others such as Navarro et al. (2010) or Vizcaíno and Aciego (2015) broaden the topics addressed, including other areas such as sexual abuse prevention. These programs have shown improvements in the areas addressed, although they do not address all the necessary areas, have been applied in unrepresentative samples given their small size (between 15 and 37 participants) and have focused mainly on the young population (between 16 and 40 years of age).

With that in mind, this research group has developed a proposal focused on a positive vision of the sexuality of people with intellectual functional diversity, aiming not only to improve knowledge and skills, but also attitudes. This is the SALUDIVERSEX program (Gil-Llario et al., 2019) which was designed to be implemented by the occupational center professionals after having received the training and guidelines to apply it. Thus, once trained, it is ensured that a sufficiently trained person remains permanently in the center, capable of responding to the problematic circumstances that may arise in relation to the experience of sexuality in the environment in which the user spends most of his/her time.

### Comparison SALUDIVERSEX-Extended Version Versus SALUDIVERSEX-Reduced Version

For all the benefits of positive affective-sexual education, as has been proven (Gil-Llario et al., 2021b; McDaniels & Fleming, 2016), the demand for occupational centers has grown exponentially in recent years. However, their day-to-day reality, aggravated by the restrictions resulting from the COVID-19 health emergency, makes it very difficult to include a large number of program sessions among the many other activities carried out by users. Thus, compared to the extended version of the SALUDIVERSEX program, which consists of 16 sessions and whose efficacy has already been proven (Gil-Llario et al., in press), a reduced version of the program was chosen in the hope of finding the same, if not better, results.

The reduced version is made up of 10 sessions that are grouped into the same 4 blocks of the original program: (I) *Introduction to sexuality,* which addresses the concept and functions of sexuality, the concept of intimacy, body image and self-esteem, and communication as an essential element in affective-sexual relationships; (II) *Self-care,* which deals with sexual hygiene, autoeroticism and sexual diversity; (III) *The self in relationship*, in which training is provided on the initiation, maintenance and rupture of affective-sexual relationships, sexual assertiveness, sexual practices and safe sex, prevention of infections and unwanted pregnancies, love and maltreatment, and prevention of sexual abuse; and (IV) *My sexuality,* which includes only one session of integration and consolidation of learning. In the reduced version, although the basic concepts, skills and attitudes of the original program have been maintained, the depth with which the modules on body image, prevention of sexually transmitted infections and contraceptive methods, and inappropriate behaviors in social networks have been addressed has been reduced. This adaptation is based on the results obtained in the evaluation of the effectiveness of the extended version of the program (Gil-Llario et al., in press) and on the feedback provided by the professionals who applied it and by the participants themselves. Thus, while the validation of the original version of the SALUDIVERSEX program reported the high starting levels of the subjects in some of these modules and the small margin for improvement they showed, the professionals who applied them and the users themselves, assessing the real characteristics and needs of the target group, considered that it was unnecessary to go so deeply into these contents.

In relation to the teaching methodology, the shorter version has limited theoretical explanations through the use of expository techniques, although visual material has continued to be used to support them (slides and short and attractive videos), activities that invite reflection and guided debates. On the other hand, playful dynamics and role-playing games have become the star technique to encourage user participation. Also, following the feedback from the participants and instructors of the extended version of the program, although the number of activities within each session is the same, the number of exercises within each session was reduced in order to better address the doubts that arose during their implementation.

In view of the above and given the importance of knowing whether these modifications have an impact on user learning, the objective of this work is to analyze the differential efficacy of both versions. If the results of the reduced version were better or at least similar to those of the extended version, we would propose the use of the reduced version given the benefits that, as has been justified, a smaller number of sessions entails.

## Method

### Participants

The total sample in the present study was 208 participants who attend two support service networks for people with ID. Participants were taken from multiple support centers within each network. To determine eligibility for the research, we established the following inclusion criteria: (1) age 18 years old or over; (2) have an Intellectual Disability as outlined by the DSM-5 criteria; and (3) have enough communication and reading skills to carry out the activities of the study. Centers of one of the support service networks for people with ID received the extended version of the SALUDIVERSEX program (*n* = 152), and centers of the other received the reduced version of the SALUDIVERSEX program (*n* = 56).

Among participants in the extended version of the program, 52% (*n* = 79) were women and 48% (*n* = 73) were men, with ages ranging from 19 to 67 years old (*M* = 36.64, SD = 10.49). The large majority of participants lived in their parents or guardians’ homes (*n* = 131, 86.2%), followed by 5.9% (*n* = 9) living in nursing home/hospital settings for people with ID, the same percentage of participants lived in community facilities with different degrees of supervision, and only 2% (*n* = 3) of participants lived alone.

Among participants in the reduced version of the program, 42.86% (*n* = 24) were women and 53.14% (*n* = 32) were men, and their ages ranged between 21 and 64 years old (*M* = 38.91; SD = 11.06). The majority lived with their parents or guardians (83.02%; *n* = 47), followed by 7.55% (*n* = 4) living in community facilities with different degrees of supervision, the same percentage of participants who lived alone, and only 1.88% (*n* = 1) of participants lived in nursing home/hospital settings for people with ID.

You can take a closer look at the characteristics of the participants according to extended versus reduced version of the program in Table 1.

**Table 1:** Baseline participant characteristics

	Extended version	Reduced version	Extended vs. reduced version
	*M* ± SD or % (*n*)	*M* ± SD or % (*n*)	*χ*^2^ or *t* test
Gender			0.34
Female	48% (73)	42.86% (24)	
Male	52% (79)	53.14% (32)	
Age	36.64 ± 10.49	38.91 ± 11.06	− 1.33
Residence type			5.09
With relatives (with parents, siblings, guardians, etc.)	86.2% (131)	83.02% (47)	
Community living (shared apartment with complete/ partial supervision)	5.9% (9)	7.55% (4)	
Nursing home/hospital setting (nursing home, congregate care, etc.)	5.9% (9)	1.88% (1)	
Independent living (alone or with others with no supervision)	2% (3)	7.55% (4)	

The validity of the results obtained in a self-reported manner in relation to the effectiveness of the two versions of the program was evaluated by obtaining sexual concerns and knowledge assessment of the participants from 2 professionals at each center (a total of 44; 34 and 10 for extended and reduced version program, respectively).

### Measures

#### Demographics

Gender and age were self-reported by the participants, whereas the professionals responsible for their care at the support services were asked about the participants’ residence type.

#### Self-Report Instrument for the Assessment of Sexual Behavior and Concerns of People with Mild Intellectual Disabilities (SEBECOMID-S; Gil-Llario et al., 2021c***)***

The SEBECOMID-S is a self-administered instrument to assess sexual behavior and concerns of people with mild ID that includes 14 questions. The questions have different response formats depending on the content: a frequency scale ranging from never to always (e.g., “How often do you use a condom when you have vaginal intercourse with your partner?”) and dichotomous questions with yes/no answers (e.g., “Have you ever masturbated?”). This instrument includes four main aspects: *sexual response*, or reaction to excitation (e.g., “When you see pictures of people you like or someone you are attracted to is near you, do you feel like touching yourself?”); *worry*, or concerns of people with ID about issues related to sex or interpersonal relationships (e.g., “Do you worry that people you like will look at you funny or misunderstand you when you show that you like them?”); *sex practices*, or sexual activities that people with ID might engage in (e.g., “Have you ever had anal intercourse?”); and *condom use*, or safe sex practices (e.g., “Do you talk your partner into using a condom?”). The reliability of the factors ranged from 0.64 to 0.72, being similar for men and women.

#### Detection of Sexual Abuse Risk Screening Scale (DSARss; Gil-Llario et al., 2020a***)***

The DSARss are a brief screening measure designed to assess the risk of experiencing sexual abuse (SA) for people with ID. The scale consists of 19 items, which are grouped into four factors: (1) the denial of the risk of SA by people in the victim's immediate environment ("Acceptance of the abuse due to affection," e.g., “My father takes care of me, so it is okay to have sexual relationships with him.”), (2) the perception of invulnerability to SA associated with places ("Denial of the risk associated with places," e.g., “It is impossible to be sexually abused in the street.”), (3) the presence of risk indicators associated with drug use or lack of parental supervision and mastery of coping skills ("Risk factors and self-protection skills," e.g., “It is better not to say anything if someone touches my privates without my consent.”), and (4) the person's knowledge about what constitutes a potential threat to personal space ("Lack of awareness of intimacy rules," e.g., “It is okay if someone I know touches my butt.”). All items are dichotomous (true or false) and include an illustration exemplifying the content of the question, in order to help people with ID understand the content of the item and provide reliable responses. Reliability analysis of the DSARss found acceptable internal consistency for the total scale (*α* = 0.51) and for the four factors (*α* ranging between 0.5 and 0.71). It could be understood that these reliability coefficients are not as high as expected due to the limitations involved in the validation of measurement instruments in this population. The poor metacognition and communication skills, the problems of reasoning, planning and abstract thinking, and the high social desirability of this group, lead us to consider lower reliability coefficients acceptable (Hartley & MacLean, 2006). Moreover, this instrument is sensitive to gender, making it suitable for the detection of AS in both men and women.

#### Inventory of Sexual Knowledge of People with Intellectual Disability (ISK-ID; Gil-Llario et al., 2021d***)***

The ISK-ID is a scale designed to be applied as a self-report measure and provides a measure of sexual knowledge across six different sexuality domains: (a) knowledge about what kind of sexual activities may be considered sexual or not depending on the context (“sexuality concepts,” e.g., “A part of the body such as the ear is sexual or not depending on the person who touches it and the situation”); (b) knowledge about how to have a positive body image and communicate sexually (“body image and sexual communication,” e.g., “If a have a beautiful body, I will have a positive body image”); (c) knowledge about the nature of different sexual practices, such as masturbation, oral sex, or vaginal and anal intercourse (“sexual practices,” e.g., “When the penis is inside the vagina or the anus, it is called intercourse”); (d) knowledge about sexual diversity (“homosexuality,” e.g., “It is wrong for two men or two women to kiss on the mouth”); (e) knowledge about how to interact with a romantic/sexual partner in the context of an intimate relationship (“dating, intimacy, and sexual assertiveness,” e.g., “Boyfriends/girlfriends are forever. Therefore, I do not break up with my boyfriend/girlfriend even if he/she wants to”); and (f) knowledge about how to prevent STIs and unwanted pregnancy (“sexual health,” e.g., “Contraceptive methods are useful to prevent unwanted pregnancies”). It is composed of 34-item that are written in an easy-to-read format and rated on a dichotomous scale (Yes/No), thus helping individuals with ID to understand the item content and provide reliable responses. It takes about 20 min to complete. With respect to its psychometric properties, reliability analysis of the ISK-ID found good internal reliability for the total scale (*α* = 0.76) and acceptable for the six factors (*α* ranging between 0.54 and 0.67). Furthermore, this instrument proved to be reliable in assessing sexual knowledge in men and women.

#### Assessment of Sexual Behavior and Knowledge of People with Intellectual Disability (ABSKID; Gil-Llario et al., 2020b***)***

The ABSKID is a 24-item other-reported instrument to be completed by professionals working with people with ID in occupational settings. The main components are: concern about the user's inappropriate or uninhibited sexual behavior (“BEH-UNINHIB,” e.g., “do you know if s/he has ever masturbated in public?”), perception of user’s knowledge about privacy and social norms (“PRIV-NOR,” e.g., “do you think s/he is aware of social norms about not letting others touch one's private body parts”), perception of user's knowledge about sexuality (“KNOW-SEX,” e.g., “do you think s/he understands the human reproduction process?”), and concerns about the user's sexuality (“CONCERN,” e.g., “are you worried that s/he won't find a partner?”). The items have a dichotomous “Yes” or “No” response format. The reliability of the factors ranged from 0.59 to 0.74, being equivalent in both genders.

### Procedure

The head of the research project (M. D. Gil-Llario) was contacted by two support service networks for people with ID given the detected needs and their interest in their users receiving an appropriate affective-sexual education adapted to their characteristics. Once the demand was confirmed, our research team got involved in the study of the needs of this group in this area of development. This project allowed us to lay the foundations for the development of an intervention program. The principal investigator held several meetings with the heads of the main support service networks to present the objectives of the project and explain how the intervention would be tested.

Once both institutions agreed to participate, 22 occupational centers (clusters) within the two institutions selected to participate in this research. To ensure sample representativeness, we followed a stratified random sampling procedure based on population density to select these centers (Lohr, 2010). We prioritized the selection of centers located in urban areas with a medium population density, and we then completed with the inclusion of centers located in areas with high and low population densities. Thus, in the final sample, around 22.1% of the participants were from cities with a population density > 500,000, around 65.3% were from cities with a population density between 10,000 and 500,000, and around 13.1% were from cities with < 10,000 inhabitants (considered rural areas). This recruitment procedure allowed us to obtain a representative sample of individuals with ID from urban and rural areas.

The users of the 17 selected occupational centers belonging to the first support service network for people with ID that contacted us became the experimental group, while the users of the 5 occupational centers belonging to the support service network that contacted us second became the wait-list group. Thus, initially, the first 17 occupational centers received the extended version of the SALUDIVERSEX program. However, at the end of this implementation, some changes were made to this version of the program with the aim of improving it and adapting it to the real context of people with ID, including changes suggested by professionals and participants. These changes were very positively received when we resumed the research after the Covid-19 pandemic since, the fact of reducing the number of sessions, facilitated the implementation of the program in post-pandemic. The contents on which, according to feedback from professionals and users and the results of the analysis of the effectiveness of the extended version of the program, were not necessary to go into such depth were eliminated, and theoretical explanations and the use of reading and writing-laden activities were reduced to a minimum. This reduced version of the SALUDIVERSEX program was implemented in the 6 centers that until then were part of the wait-list group.

However, regardless of whether they received the extended or reduced version of the program, all participants were assessed twice: before the implementation of the intervention (pre-test) and two weeks after finishing the program (post-test). As for the assessment procedure, two members of the research team with extensive experience in the assessment and treatment of people with ID carried out the data collection. Each participant was assessed individually in a private and quiet room of the support service. Only the participant and one of the evaluators were present while completing the assessment and evaluators kept their distance from participants to respect their privacy and create a comfortable situation. Participants were given a brief explanation about how to complete the assessment tools and, if needed, support was provided while they were filling them in (e.g., explaining the meaning of a word). The two professionals from each center also received the necessary copies of the questionnaire to be filled in about each user, and were given a couple of weeks to complete them (adjusting to their workload), during which time they could contact the principal investigator if they had any doubts.

We consulted participants’ educational supervisors in order to assess their reading skills. Participants were excluded from the research only if they did not have high enough comprehension and communication skills to carry out the assessment procedure with the researcher’s assistance. Participants were also excluded from the study if their guardians did not sign the informed consent.

The implementation of the program was carried out under the same conditions regardless of the version of the program. It was applied by the caretakers of the support services in groups of no more than 10 people. Before the implementation of the program, these caretakers received extensive instruction on how to apply the program and were provided all required materials. The sessions were held in large spaces, set up with tables and chairs in order to provide a work area for participants, as well as another open area where role-playing could take place. Privacy was also ensured so that participants could express themselves freely without fear of being overheard by people outside the group. Prior to the implementation of the program, all participants agreed to maintain the privacy of what was said by their peers during the sessions. Sessions occurred once a week. Each session began with a set of “pre-questions” which focused on participants’ previous knowledge. These “pre-questions” were followed by the development section, which included activities and explanations. Special attention was paid to the adaptation of the contents according to the autonomy and abilities of each participant. Finally, at the end of each session a summary sheet was presented with the main takeaways from the session. This summary sheet was shown again at the beginning of the next session and periodically throughout the program in order to help participants consolidate what they had learned.

All participants filled out an informed consent form and were informed about the confidentiality of their answers and the purpose for which the data were to be used. The study complied with the ethical principles of the Declaration of Helsinki and was approved by the Experimental Research Ethics Committee of the University of Valencia (Spain).

### Statistical Analyses

#### Descriptive Analysis

We obtained descriptive statistics of the sample, i.e., means and SDs for numerical variables and percentages for categorical variables by groups at pre-test and at post-test. Inferential statistics of the outcomes were calculated comparing both groups using chi-squares for categorical variables and independent sample *t* tests for numerical variables after examining normality assumptions.

#### Efficacy of the Intervention

Effect sizes (ESs) of the raw mean differences were obtained, using the standardized mean difference, *d* (Hedges, 1981), experimental vs. control, controlling for baseline differences between groups (Becker, 1988). Following Cohen’s classification the magnitude of the standardized *d* value can be interpreted as 0.25, 0.5, and 0.8 for small, median, and large effects on the outcomes of interest. ESs were calculated using an effect size calculator (Huedo-Medina & Johnson, 2011).

Multilevel models were conducted to evaluate the efficacy of the intervention controlling for the dependence of the participants who belong to the same occupational center, i.e., individuals nested within the same cluster. One level of random effects, occupational center-random effects was included in the model to account for the dependence within the occupational centers. The intervention effect at post-test was defined as a difference in changes from baseline between the intervention and control group. Goodness of fit of the models was compared using Akaike's Information Criterion (AIC), Schwarz's Bayesian Criterion (BIC) and -2 Restricted Log Likelihood. Mixed-effects regression models were ran using MIXED macro from SPSS v.24 (Hox et al., 2010; McCulloch and Searle, 2001).

For all statistical tests, a *p* value of less than 0.5 will be considered statistically significant, which will reject the null hypothesis. If the *p* value is above 0.5, then the null hypothesis will be accepted. Missing data were treated with a complete case analysis.

## Results

### Efficacy of Reduced Versus Extended Version of the Program SALUDIVERSEX

#### Sexual Behavior and Concerns

Regardless of the version of the program in which they participated, users generally obtained higher mean scores after participating in the program (post-test) on all sexual behaviors and concerns assessed, and effect sizes comparing both versions were in favor of the reduced program (Table 2). We observed a significant program effect on all factors related to their sexual behavior (Table 3), users who participated in the reduced version presented a significantly greater increase compared to those in the extended version after controlling for cluster dependence and pre-test (*Sexual response*: *β*_10_ = − 0.46 ± 0.19, *p* = .034; *Sex practices*: *β*_10_ = − 0.52 ± 0.23, *p* = .037; *Use condom: β*_10_ = − 1.56 ± 0.59, *p* = .017). However, with respect to their concerns (*“worry”*), although this increase is also greater in the group that participated in the reduced version, these differences were not significant (*β*_10_ = − 0.57 ± 0.28, *p* = .06).

**Table 2:** Effect sizes of comparisons extended vs. reduced version program

		Assessment time	Extended version	Reduced version	Extended vs. reduced version			
			Mean (SD)	Mean (SD)	*d*	95% CI^2^		ICC^1^
Sexual behavior and concerns	*Sexual response*	Pre-test	1.2 (1.16)	1.53 (1.14)	− 0.18	− 0.5	0.15	0.07
		Post-test	1.08 (1.08)	1.61 (1.11)				0.07
	*Worry*	Pre-test	1.1 (1.05)	1.44 (0.97)	− 0.13	− 0.46	0.19	0.16
		Post-test	1.27 (1.02)	1.73 (1.3)				0.19
	*Sex practices*	Pre-test	1.19 (1.27)	2.22 (1.72)	− 0.10	− 0.23	0.43	0.09
		Post-test	1.53 (1.28)	2.51 (1.64)				0.12
	*Use condom*	Pre-test	4.76 (3.64)	7.25 (3.84)	0.00	− 0.33	0.33	0.09
		Post-test	5.96 (3.49)	7.53 (.73)				0.11
Risk of experiencing SA	*Acceptance of the abuse due to affection*	Pre-test	0.23 (0.61)	0.27 (0.79)	0.14	− 0.19	0.46	0.02
		Post-test	0.20 (0.55)	0.12 (0.33)				0.12
	*Denial of the risk associated with places*	Pre-test	1.53 (1.16)	1.33 (1.07)	0.23	0.09	0.56	0.17
		Post-test	1.11 (1.13)	0.69 (0.93)				0.26
	*Risk factors and self-protection skills*	Pre-test	1.86 (1.25)	7.02 (1.25)	0.06	− 0.26	0.39	0.78
		Post-test	1.82 (1.22)	6.9 (1.7)				0.78
	*Lack of awareness of intimacy rules*	Pre-test	1.01 (1.01)	1.47 (1.7)	− 0.12	− 0.45	0.2	0.07
		Post-test	0.69 (0.75)	1.14 (0.79)				0.22
	*Total score*	Pre-test	4.63 (1.99)	10.14 (2.58)	0.05	− 0.28	0.38	0.6
		Post-test	3.81 (2.01)	8.94 (2.03)				0.65
Sexual knowledge	*Sexuality concepts*	Pre-test	1.73 (1.34)	2.21 (1.28)	0.27	− 0.05	0.59	0.08
		Post-test	2.71 (1.18)	2.8 (1.23)				0.34
	*Body image and sexual communication*	Pre-test	2.57 (1.44)	3.39 (1.25)	0.58	0.25	0.91	0.23
		Post-test	3.78 (1.28)	3.71 (1.22)				0.14
	*Sexual practices*	Pre-test	4.33 (1.87)	4.59 (1.56)	− 0.21	− 0.53	0.12	0.13
		Post-test	5.15 (1.73)	5.61 (1.6)				0.06
	*Homosexuality*	Pre-test	2.33 (0.86)	2.48 (0.81)	− 0.03	− 0.35	0.29	0.15
		Post-test	2.44 (0.79)	2.61 (0.64)				0.25
	*Dating, intimacy and assertiveness*	Pre-test	4.98 (2.04)	4.87 (1.98)	− 0.35	− 0.67	− 0.02	0.07
		Post-test	6.04 (1.80)	6.61 (1.69)				0.1
	*Sexual health*	Pre-test	3.67 (1.48)	4.82 (1.73)	0.35	0.03	0.68	0.16
		Post-test	4.07 (1.26)	4.67 (0.74)				0.43
	*Total score*	Pre-test	20.57 (4.70)	22.73 (5.04)	0.29	− 0.03	0.62	0.21
		Post-test	24.98 (4.88)	26 (5.11)				0.22
User’s knowledge and sexual behavior reported by professionals	*BEH-UNINHIB*	Pre-test	0.56 (0.94)	0.34 (0.73)	0.2	− 0.11	0.52	0.09
		Post-test	0.46 (0.79)	0.11 (0.38)				0.1
	*PRIV-NOR*	Pre-test	4.81 (0.60)	3.96 (0.99)	− 0.99	− 1.32	− 0.66	0.32
		Post-test	4.82 (0.54)	4.98 (.14)				0.05
	*KNOW-SEX*	Pre-test	5.71 (2.58)	7.79 (2.21)	0.54	0.22	0.86	0.38
		Post-test	7.52 (1.80)	8.15 (1.82)				0.18
	*CONCERN*	Pre-test	2.54 (1.09)	1.23 (1.29)	− 1.62	− 1.98	− 1.27	0.46
		Post-test	0.86 (1.01)	1.35 (1.48)				0.33

^1^These are the Intraclass Correlation Coefficient from the unconditional models

^2^95% CI = 95% Confidence Interval

**Table 3:** Multilevel model comparing the extended vs. reduced version of the program in relation to sexual behavior and concerns

	Sexual response		Worry		Sex practices		Use condom	
	Estimate (SE)	*p*	Estimate (SE)	*p*	Estimate (SE)	*p*	Estimate (SE)	*p*
Fixed-effects								
*β*_00_ (Intercept)	1.07 (0.2)	< .001	1.52 (0.27)	< .001	1.22 (0.24)	< .001	5.74 (0.69)	< .001
*β*_10_ (Group)^2^	− 0.46 (0.19)	.034	− 0.57 (0.28)	.06	− 0.52 (0.23)	.037	− 1.56 (0.59)	.017
*β*_20_ (Pre-test)^1^	0.39 (0.07)	< .001	0.26 (0.08)	.001	0.63 (0.59)	< .001	0.41 (0.07)	< .001
Random-effects								
*τ*_00_ (Intercept)	0.04 (0.02)	.492	0.19 (0.09)	.047	0.06 (0.06)	.373	0.05 (0.45)	.907
*ρ*_logit_	0.19 (0.02)	< .001	0.93 (0.1)	< .001	1.09 (0.13)	< .001	10.06 (1.16)	< .001
Fit indexes								
AIC	519.7		535.83		498.83		896.08	
BIC	526.06		542.22		505		902.35	
LogLik	515.7		531.83		594.83		892.08	

^1^All models are adjusted for pre-test

^2^Multilevel models. Positive scores greater than 0 means the favoring the extended version of the program

#### Risk of Experiencing SA

As we can see in Table 2, both users who participated in the reduced version of the program as those who participated in the extended version obtain lower scores in the post-test than in the pre-test in all factors (negative factors). Effect sizes were in favor of the reduced version of the program in all variables, except for *lack of awareness of intimacy rules.* The comparison between both versions of the program in the multilevel model controlling for pre-test and cluster dependence (Table 4) shows a significant improvement among participants of the reduced version compare to the longer version on the *risk factors and self-protection skills* (*β*_10_ = − 3.90 ± 0.53, *p* < .001). There was a significant decrease in the total score of *risk of experiencing SA* among the participants of the reduced compare to the long version (*β*_10_ = 3.95 ± 0.64, *p* < .001). In the case of the *lack of awareness of intimacy rules*, although the difference in improvement between the two versions is not significant, it is very close to being so, in favor of the reduced version (*β*_10_ = − 0.04 ± 0.2, *p* = .059).

**Table 4:** Multilevel model comparing the extended vs. reduced version of the program regarding with the dimensions of the risk of experiencing SA

	Acceptance of the abuse due to affection		Denial of the risk associated with places		Risk factors and self-protection skills		Lack of awareness of intimacy rules		Total scale_risk of experiencing SA	
	Estimate (SE)	*p*	Estimate (SE)	*p*	Estimate (SE)	*p*	Estimate (SE)	*p*	Estimate (SE)	*p*
Fixed-effects										
*β*_00_ (Intercept)	0.05 (0.08)	.554	0.47 (0.28)	.011	5.28 (0.61)	< .001	1.02 (0.19)	< .001	3.98 (0.55)	< .001
*β*_10_ (Group)^2^	0.09 (0.09)	.351	0.49 (0.30)	.12	− 3.9 (0.53)	< .001	− 0.4 (0.2)	.059	3.95 (0.64)	< .001
*β*_20_ (Pre-test)^1^	0.26 (0.05)	< .001	0.12 (0.07)	.087	0.23 (0.07)	.002	0.08 (0.04)	.069	0.27 (0.06)	< .001
Random-effects										
*τ*_00_ (Intercept)	0.007 (0.01)	.599	0.24 (0.12)	.042	0.36 (0.16)	.028	0.09 (0.05)	.063	0.8 (0.39)	.040
*ρ*_logit_	0.22 (0.03)	< .001	0.91 (0.10)	< .001	1.42 (0.16)	< .001	0.49 (0.05)	< .001	3.51 (0.39)	< .001
Fit indexes										
AIC	260.72		537.65		620.83		418.54		769.2	
BIC	267.12		544.05		627.24		424.93		775.56	
LogLik	256.72		533.65		616.83		414.54		765.2	

^1^All models are adjusted for pre-test

^2^Multilevel models. Positive scores greater than 0 means the favoring the extended version of the program

#### Sexual Knowledge

When analyzing the level of sexual knowledge, we observed a tendency for participants in both groups to obtain higher mean scores after receiving the intervention. ESs comparing the extended and reduced version were low for all factors, except for the factor about *body image and sexual communication*, whose effect was medium and significant in favor of the extended version (*d* = 0.58; 95% CI = 0.25, 0.91) (Table 2). This similar trend of both groups is reflected in the results of the multilevel model, the participants of the reduced version show a greater range of improvement, but they were not significant in their knowledge of the different variables measured (Table 5).

**Table 5:** Multilevel model comparing the extended vs. reduced version of the program regarding with the domains of the sexual knowledge

	Sexuality concepts		Body image and sexual communication		Sexual practices		Homosexuality		Dating, intimacy, and sexual assertiveness		Sexual health		Total scale_Sexual knowledge	
	Estimate (SE)	*p*	Estimate (SE)	*p*	Estimate (SE)	*p*	Estimate (SE)	*p*	Estimate (SE)	*p*	Estimate (SE)	*p*	Estimate (SE)	*p*
Fixed-effects														
*β*_00_ (Intercept)	2.46 (0.37)	< .001	3.14 (0.36)	< .001	4.28 (0.43)	< .001	2.01 (0.21)	< .001	5.05 (0.43)	< .001	3.37 (0.29)	< .001	12.14 (1.9)	< .001
*β*_10_ (Group)^2^	0.02 (0.41)	.959	0.22 (0.32)	.5	− 0.34 (0.35)	.34	− 0.14 (0.15)	.379	− 0.59 (0.34)	.1	− 0.34 (0.19)	.102	− 0.09 (1.21)	.94
*β*_20_ (Pre-test)^1^	0.17 (0.06)	.008	0.17 (0.07)	.015	0.29 (0.07)	< .001	0.24 (0.07)	< .001	0.31 (0.06)	< .001	0.28 (0.05)	< .001	0.41 (0.07)	< .001
Random-effects														
*τ*_00_ (Intercept)	0.51 (0.015)	.015	0.23 (0.13)	.071	0.19 (0.16)	.223	0.04 (0.03)	.285	0.18 (0.16)	.267	0.01 (0.05)	.79	3.56 (1.98)	.071
*ρ*_logit_	0.88 (0.11)	< .001	1.32 (0.15)	< .001	2.43 (0.27)	< .001	0.46 (0.05)	< .001	2.59 (0.29)	< .001	1.08 (0.12)	< .001	12.55 (1.7)	< .001
Fit indexes														
AIC	459.59		577.49		688.49		374.44		721.88		506.99		714	
BIC	465.65		583.81		694.85		380.69		728.29		513.24		719.67	
LogLik	455.59		573.49		684.49		370.44		717.88		502.99		710	

^1^All models are adjusted for pre-test

^2^Multilevel models. Positive scores greater than 0 means the favoring the extended version of the program

### The Practitioner's Perspective as a Measure of Validity of the Effectiveness of the Reduced and Extended Version of the Program

In order to verify the results obtained on the efficacy of the educed vs. extended version of the SALUDIVERSEX program from participants’ self-reported information, professionals in direct contact with them provided hetero-reported information on the users' knowledge and sexual behaviors before and after the intervention.

After the intervention, professionals' concern about users’ inappropriate or uninhibited sexual behavior decreased, regardless of which version of the program they had received. However, the concerns about the misinterpretation of their sexuality and their risk of possessing misconceptions or experiencing loneliness and/or sexual abuse (*CONCERN*) decreased for the group receiving the extended version and went up for those receiving the reduced version. Likewise, professionals stated that participants in both versions increased their knowledge about privacy and social norms, although in the extended version it was minimal, as well as their knowledge about sexuality after participating in the program. The ESs comparing extended vs. reduced version of the program showed a large and significant effect for the domains related to the professionals' perception of user’s knowledge about privacy and social norms (*d* = − 0.99; 95% CI = − 1.32, − 0.66) and about the interpretation of their sexuality and their risk of possessing misconceptions or experiencing loneliness and/or sexual abuse (*d* = − 1.62; 95% CI = − 1.98, − 1.27). The ESs showed moderate and significant effect for the professionals' perception of user’s knowledge about sexuality (*d* = 0.54; 95% CI = 0.22, 0.86) (Table 2).

The group differences also reached significance in the multilevel model in all domains (*BEH-UNINHIB*: *β*_10_ = 0.21 ± 0.07, *p* = .012; *PRIV-NOR*: *β*_10_ = − 0.48 ± 0.08, *p* < .001; *CONCERN: β*_10_ = − 1.35 ± 0.21, *p* < .001), with the exception of the user's knowledge about sexuality (*KNOW-SEX: β*_10_ = 0.56 ± 0.39, *p* = 0.164) (Table 6). According to the direction of the results obtained, it seems to be the users who received the reduced version who increase to a greater extent their knowledge about privacy and social norms and their concerns about having misconceptions or experiencing loneliness and/or sexual abuse, after the intervention according to the professionals, and they also reduced more their concerns about users’ inappropriate or uninhibited sexual behavior.

**Table 6:** Multilevel model extended vs. reduced version program in relation to user’s knowledge and sexual behavior reported by professionals

	BEH-UNINHIB		PRIV-NOR		KNOW-SEX		CONCERN	
	Estimate (SE)	*p*	Estimate (SE)	*p*	Estimate (SE)	*p*	Estimate (SE)	*p*
Fixed-effects								
*β*_00_ (Intercept)	− 0.14 (0.06)	.043	3.41 (0.15)	< .001	3.54 (0.43)	< .001	0.53 (0.18)	.009
*β*_10_ (Group)^2^	0.21 (0.07)	.012	− 0.48 (0.08)	< .001	0.56 (0.39)	.164	− 1.35 (0.21)	< .001
*β*_20_ (Pre-test)^1^	0.71 (0.03)	< .001	0.39 (0.03)	< .001	0.59 (0.04)	< .001	0.67 (0.06)	< .001
Random-effects								
*τ*_00_ (Intercept)	0.008 (0.007)	.279	0.01 (0.007)	.068	0.43 (0.19)	.024	0.08 (0.05)	.123
*ρ*_logit_	0.12 (0.01)	< .001	0.09 (0.009)	< .001	1.03 (0.12)	< .001	0.59 (0.07)	< .001
Fit indexes								
AIC	151.06		101.14		568.06		447.99	
BIC	157.45		107.53		574.45		454.38	
LogLik	147.06		97.14		564.06		443.99	

^1^All models are adjusted for pre-test

^2^Multilevel models. Positive scores greater than 0 means the favoring the extended version of the program

## Discussion

To analyze the differential effectiveness of the two versions of the program, rather, the extended vs. the reduced version, three indicators of change from the users themselves and one from the professionals who work with them on a daily basis have been taken into consideration. The indicators of effectiveness from the users' point of view have been sexual concerns and behavior, risk of experiencing sexual abuse, and level of knowledge acquired after the implementation of the program. In the case of professionals, the concerns they have in relation to the level of disinhibition of users’ behavior, and the knowledge they perceive users have about privacy rules and about sexuality in its various aspects, have been assessed.

With regard to sexual concerns and behavior, in all the factors analyzed (sexual response, concerns about issues related to sexuality or interpersonal relationships, sexual practices and condom use), the reduced version obtains a greater range of improvement, although in the case of concerns about aspects related to sexuality or interpersonal relationships this difference between the two versions is not significant. In other words, with 10 sessions, users are able to obtain the self-knowledge necessary to know their sexual response, improve their sexual practices and acquire basic skills of self-protection by performing safe sex. This could be due to the fact that in the reduced version of the program these aspects were substantially changed. The information provided regarding some partner sexual practices (e.g., anal sex) was reduced and more information was provided on autoerotic practices, which are more frequent in this group. In addition, the sexual response was explained on the basis of masturbation, which may have helped to understand it better. Theoretical content related to some less common STIs (e.g., syphilis) and some contraceptive methods with low frequency of use in this group (e.g., IUD) was also reduced, devoting more attention to the use of condoms. Also, the main concepts were always accompanied by visual material, and the activities were reformulated to emphasize self-pleasure and the use of condoms. This remodeling, eliminating content that does not allow to focus on what is important, seems to facilitates access to knowledge for this group, which, as several studies show, needs to address these areas. This can be seen in the study of Jahoda and Pownall (2014) who found that people with ID present misconceptions about sexual practices, routes of transmission of sexually transmitted diseases and contraception, in addition to a low level of socio-affective skills. Also Graff et al. (2018) detected the need to educate on aspects related to sexual hygiene, such as the need to have gynecological checkups, and on couple relationships. Gil-Llario et al. (2018), for their part, also concluded that this is a social group with highly biased information, relatively low condom use, and a high level of submissiveness and dependence on third parties, which makes them a population more vulnerable to sexual abuse.

Regarding the risk of sexual abuse, the two versions of the program, both the reduced and extended versions, improve the protection of users, with lower post-test scores on all factors. That is, after the intervention, the acceptance of abuse due to affection decreases (that is, tolerance of abuse when it comes from a family member or person close to you), the denial of risk associated with places decreases (that is, they are aware of the risk of driving on dark, little-traveled streets) and the awareness of privacy norms improves (that is, they understand what kind of actions involve a vulnerability to their privacy and, to that extent, an abuse). Improving these aspects is of vital importance, given the high prevalence of sexual abuse in this group, mostly perpetrated by acquaintances (friends, partners or family members) (Mitra et al., 2016; Tomsa et al., 2021; Verdugo et al., 2002); as well as, because they tend to believe that other people can make decisions about their sexual experiences and to have positive or indifferent feelings toward sexual abuse, compared to the population without ID (McCabe et al., 1994). In this sense, having reduced the number of sessions while improving the results in abuse prevention is particularly relevant. The main changes with respect to the extended version of the program were the structuring of the session. Purely theoretical content was reduced by replacing it with clear examples, and the explanation was based on understanding the existence of different levels of social interaction (different levels of intimacy) and on addressing four specific questions (what, who, to whom and where). This made it possible to transmit the information in a more orderly fashion.

There are no significant differences between the two versions in the knowledge of sexuality. This is also an endorsement that reducing the number of sessions has not resulted in a decrease in the amount of relevant knowledge conveyed through the program. Work has continued to the point that course participants have assimilated the most relevant basic aspects, such as the concept of sexuality, communication as a fundamental tool for sexual relations, how and where to perform basic sexual practices safely, the concept of intimacy and assertiveness, having also acquired healthy attitudes of tolerance toward non-majority sexual orientation options, that is, prevention of homophobia mainly. In fact, in the reduced version there have been more notable improvements in all the scales, although, as indicated, these improvements do not reach the level of statistical significance. Such improvements are not shown, as is evident, only in those contents that were suppressed for the reasons given in the introduction, such as body image.

In summary, and observing all the data as a whole, we can see that both the extended and the reduced version obtain higher average scores in the post-test of all the factors, which confirms the effectiveness of both versions.

Finally, as regards the professional's perspective as a measure of validity of the program's effectiveness, previous research has shown that there is some congruence between the perception of professionals and that of users in various aspects related to sexuality and the quality of life of the latter (Caballero-Gascón et al., 2018). Thus, the role played by the professionals as the main mediators of their affective-sexual education (López, 2011), and the day-to-day coexistence with the users of the centers where they work, justifies their being reliable sources of information (Gil-Llario et al., 2021a). Regarding our findings, in the present research we see that professionals, after the application of the extended version compared to the reduced version, are still more concerned about the uninhibited behaviors that users may exhibit. That is, they feel that the shorter version has provided them with the knowledge they need and, in that sense, they are less concerned that they may engage in inappropriate behavior. On the contrary, they believe that the privacy standards acquired and the concerns of these users are greater in the reduced version. But, if we look at the overall data in the reduced version, the fear of uninhibited and inappropriate behaviors decreases and knowledge about privacy and sexuality norms in general increases, all in line with expectations. However, the short version does not reduce their concerns as much as the extended version does, probably because they understand that an appropriate intervention, regardless of whether it obtained, as has been the case, the expected results, should be maintained in the form of follow-up for a longer period of time. Professionals working with this group could be a resource to ensure the fulfillment of their right to long-term sexuality information (BOE, Ley 2/2010). However, some professionals do not respond adequately to the doubts of this group (Frawley & Wilson, 2016) or tend not to provide this education if they have not received training on sexuality (Schaafsma et al., 2014). For this reason, it is necessary to train professionals, thus promoting an affective-sexual education based on scientific knowledge and unbiased training (López, 2011).

Our findings show that both interventions have been shown to be effective. However, the reduced version of the program is more effective and efficient, given the better results obtained in most of the indicators. Improvements in sexual behavior, users' level of knowledge, as well as in the prevention of sexual abuse are noteworthy. To the extent that it is difficult for the managers of occupational centers to integrate extra activities with the permanent ones, this reduced version, offering the same or even better results in a shorter intervention time, seems the most advisable option.

## Data Availability

The data that support the findings of this study are available from the corresponding author, OFG, upon reasonable request.
